# Factors Impacting Fall Severity in Hospitalized Patients: A Retrospective Cohort Study

**DOI:** 10.3390/jcm13102827

**Published:** 2024-05-10

**Authors:** Sen-Yung Liu, Yu-Kai Yang, Chew-Teng Kor, Yi-Wei Sun, Hsin-Yu Wang, Yuan-Ting Yang, Ming-Chih Chou

**Affiliations:** 1 Institute of Medicine, Chung Shan Medical University, Taichung 402367, Taiwan; 88925@cch.org.tw; 2 Department of Physical Medicine and Rehabilitation, Changhua Christian Hospital, Changhua 500209, Taiwan; 182968@cch.org.tw; 3 Big Data Center, Changhua Christian Hospital, Changhua 500209, Taiwan; 179297@cch.org.tw; 4 Department of Pharmacy, Changhua Christian Hospital, Changhua 500209, Taiwan; 183551@cch.org.tw (Y.-W.S.); 121437@cch.org.tw (H.-Y.W.); 156393@cch.org.tw (Y.-T.Y.); 5 Division of Thoracic Surgery, Department of Surgery, Chung Shan Medical University Hospital, Taichung 402367, Taiwan; 6 School of Medicine, Chung Shan Medical University, Taichung 402367, Taiwan; 7 Department of Family and Community Medicine, Chung Shan Medical University Hospital, Taichung 402367, Taiwan

**Keywords:** fall-risk-increasing drugs (FRIDs), severity of fall, hospitalized patients

## Abstract

**Objectives**: This retrospective case-controlled study aimed to evaluate the association between the severity of fall-related injuries and fall-risk-increasing drugs (FRIDs) in hospitalized patients. **Methods**: Data were collected from Changhua Christian Hospital, Taiwan, of all adult inpatients who experienced falls between January 2017 and December 2021, and were divided into two groups based on whether they sustained severe fall-related injuries. Retrospective data that may affect the severity of fall-related injuries and the use of FRIDs were investigated. **Results**: Among 1231 documented cases of falls, 26 patients sustained severe fall-related injuries. Older patients and those with osteoporosis were more susceptible to more severe injuries from a fall. The use of mobility aids and osteoporosis medications showed protective effects against fall injuries. No significant association was observed between fall-related injuries and comorbidities or FRIDs. Multivariate analysis confirmed the inverse correlation between the use of mobility aids, osteoporosis medications, and fall severity. Patients with osteoporosis exhibited significantly higher odds of sustaining more severe injuries with a fall (odds ratio = 3.02, 95% confidence interval: 1.21–7.53). **Conclusions**: This study highlights the importance of addressing risk factors associated with fall severity among hospitalized patients. Providing mobility aids to persons at greater risk.

## 1. Introduction

Falls and fall-related injuries are significant health problems worldwide [1,2], and result in a considerable healthcare burden [3]. The World Health Organization defined a fall as an event that results in a person coming to rest inadvertently on the ground or floor or other lower level [4]. According to the Taiwan Ministry of Health and Welfare, the hospitalization expenses for each patient who has fallen and requires admission range from TWD90,000 to 130,000. Around 30% to 50% of falls result in minor injuries, like bruises or cuts, while 5% to 10% of falls lead to more severe injuries such as wrist and hip fractures, or traumatic brain injuries [5]. Falls and fall-related injuries stand as prominent contributors to diminished self-care capacity and restricted engagement in social and physical pursuits [6]. Fear of falling also leads to loss of functional independence, and poor quality of life [7].

Risk factors for falling include previous falls; strength, gait, and balance impairments; and use of specific medications [6]. In addition to physical or cognitive dysfunction, several studies have shown that polypharmacy and fall-risk-increasing drugs (FRIDs) are highly associated with falls [8,9,10,11]. FRIDs include antipsychotics [3,11,12,13], anxiolytics [3,11,12,13], sedatives [3,11,13], antidepressants [11,12,13], narcotics [3,11,13,14], antiepileptics [11,13,14], cardiovascular medications [3,9,11,13], non-steroidal anti-inflammatory drugs (NSAIDs) [3,11,14], contact laxatives [11,14], and proton pump inhibitors [11,14]. A previous study revealed that the use of more CNS-active medications is significantly associated with a higher risk of falls [11]. Adverse effects commonly induced by medications, such as unsteadiness, impaired alertness, and dizziness, increase the risk of falling [6].

Despite the wealth of literature addressing the association between FRIDs and fall incidence, few studies have delved into the specific ramifications of FRID use on the severity of fall-related injuries. This critical gap in knowledge warrants further investigation to comprehensively understand the extent of harm posed by FRIDs in the context of falls, thereby informing clinical decision-making and preventive strategies aimed at reducing fall-related morbidity and mortality. This retrospective case-controlled study aimed to evaluate associations between the severity of fall-related injuries and the use of FRIDs in hospitalized patients.

## 2. Materials and Methods

### 2.1. Study Design and Data Collection

This study is a retrospective case–control study. Data were obtained from Changhua Christian Hospital, a prominent medical center in Taiwan. Patients included were all adult inpatients who had encountered a fall between January 2017 and December 2021. Exclusion criteria include patients without demographic data, duplication cases, and underage individuals. To investigate potential risk factors of fall-related injuries, we extracted patient demographic information, comorbidities, laboratory data, and details regarding the fall-risk-increasing drugs (FRIDs) from the medical records for analysis.

### 2.2. Definitions of Falls and Fall-Related Injuries

A fall was defined as an event that makes a person rest accidentally on the ground or floor, or other lower level [4]. In this trial, ‘no injury’ refers to cases classified as having either no injuries or minor injuries (levels 0–2), while ‘injury’ encompasses levels ranging from severe injury to death (levels 3–5).

Based on the Taiwan Clinical Performance Indicator [15], fall-related injuries were classified as:

1. Level 0: No physical injury detected.
2. Level 1: Minor bruises or abrasions that only require minor treatment without health professional assistance.
3. Level 2: Wounds, bruises, sprains, and cuts requiring a medical/health professional examination, such as physical examination, laboratory tests, and radiography. Subsequently, bandaging, suturing, and administering 1–2 doses of medication may be implemented to promote healing and recovery.
4. Level 3: In addition to requiring extra visits, evaluations, or observations, surgical procedures or hospitalization may also be necessary. In cases such as fractures or pneumothorax, extended hospital stays may be required for proper treatment and care.
5. Level 4: Permanent handicap or long-term functional impairment, such as limb disabilities or brain injuries.
6. Level 5: Death.

### 2.3. Ethical Considerations

The study protocol underwent rigorous review and received approval from the Institutional Review Board (IRB) Committee A of Changhua Christian Hospital in Taiwan (IRB Number: 220818), documented on 31 August 2022. Given the retrospective nature of the study and the de-identification of all subjects’ private data, the IRB waived the requirement for signed informed consent. All clinical investigations strictly adhered to the guidelines outlined in the 2013 Declaration of Helsinki.

### 2.4. Data Analysis

Several covariates representing demographic and clinical background including age, gender, education level, body mass index (BMI), comorbid disease at admission, fall risk assessment at admission, dependence, laboratory data at admission, visual and hearing evaluation, admission diagnosis, and the use of FRIDs were selected (Table 1 and Table 2).

Continuous variables that were normally distributed were compared with Student’s *t*-test, and the Mann–Whitney U test was used to compare non-normally distributed data. Categorical variables were compared using the chi-squared test and Fisher’s exact test, as appropriate. To identify the risk factors for injuries associated with falls, a multivariate adjustment model was used. A crude/univariate logistic regression model was used to calculate odds ratios (OR) and 95% confidence intervals (CI). Using multivariate logistic regression, we calculated the adjusted OR (aOR) and 95% CI, adjusting for demographic and clinical data with a *p*-value of 0.05 in Table 1. All statistical analyses were carried out using SAS version 9.4 (SAS Institute Inc., Cary, NC, USA). All analyses were 2-tailed, and a value of *p* < 0.05 was considered statistically significant.

## 3. Results

**Figure 1:**
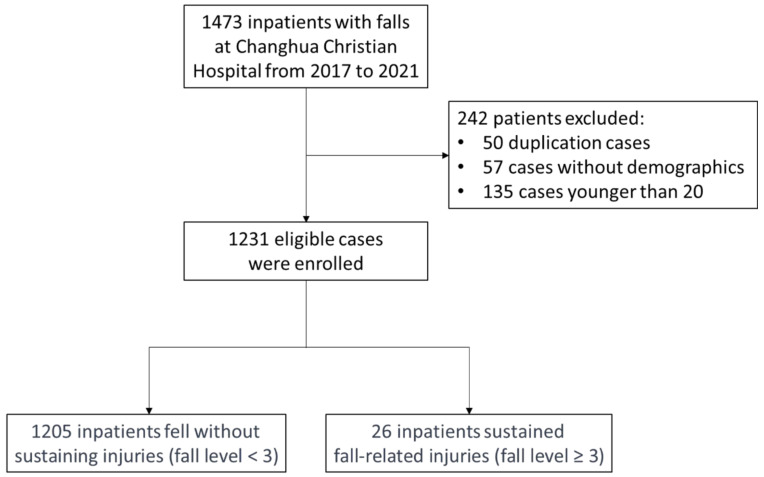
Flow diagram of patient inclusion.

A total of 1473 inpatients experienced falls at Changhua Christian Hospital from 2017 to 2021, and 242 patients were excluded. The current study analyzed 1231 fully documented cases of falls, comprising 26 patients with severe fall-related injuries (fall level ≥ 3) and 1205 patients without severe fall-related injuries (fall level < 3), as illustrated in Figure 1. The analysis of factors and the influence of FRIDs on the severity of falls is summarized in Table 1 and Table 2.

The results showed that older patients were more susceptible to falls with greater injury severity, with a mean age of 71.9 in the injury group (level ≥ 3) and 64.7 in the no fall injury group (level < 3). Within the fall injury group, 23.08% of individuals were diagnosed with osteoporosis (with or without drug therapy), while the prevalence was 8.8% in the no fall injury group. This indicates that persons with osteoporosis were also more likely to have higher severity injuries with falls. The use of mobility aids was associated with less severe injuries with falls, since the percentages of patients using mobility aids were 61.5% and 78.4% with a statistically significant difference between the two groups. No significant association was observed between fall-related injuries and comorbidities, dependence, laboratory data, or visual or hearing impairment (Table 1).

There are no statistically significant differences between the two groups in using FRIDs, including CNS-active medications (opioids, antipsychotics, anxiolytics, hypnotics and sedatives, antidepressants, antiepileptics), cardiovascular medications (vasodilators, antihypertensives, diuretics, beta-blocking agents, calcium channel blockers, renin-angiotensin system inhibitors, alpha-adrenoreceptor antagonists), and others (anti-Parkinson drugs/anticholinergic agents, diabetes medications, NSAIDs, contact laxatives, proton pump inhibitors) or not (*p* > 0.05), which may indicate that FRIDs use did not affect the severity of fall injuries.

In our study, we performed two adjustment models to assess the association between the exposure variables and severe fall injury (level ≥ 3) while controlling for potential confounders. The first adjustment model included variables such as age, mobility aid use, hemoglobin level, potassium (K) and sodium (Na) levels, hearing impairment, sleep disorder, and osteoporosis status. The second adjusted model included the same variables but replaced osteoporosis status with osteoporosis medication status (Table 3). The first multivariate adjustment model showed that the use of mobility aids was inversely correlated with the severity of fall injuries (OR = 0.40, 95% CI: 0.19–0.88). Patients with osteoporosis were significantly more likely to have a higher severity injury with a fall (OR = 3.02, 95% CI: 1.21–7.53). The second model also showed an inverse correlation with severe fall-related injuries (OR = 0.40, 95% CI: 0.18–0.87). Patients diagnosed with sleep disorder (OR = 3.24, 95% CI: 0.01–10.4) and osteoporosis without drug therapy (OR = 4.23, 95% CI: 1.44–12.43) may related to severe fall-related injuries. These findings underscore the importance of age, the use of mobility aids, and the treatment of osteoporosis in reducing the severity of fall-related injuries in hospitalized patients.

## 4. Discussion

To the best of our knowledge, this is the most comprehensive study evaluating the correlation between the severity of fall-related injuries and FRIDs. Our study includes a broader range of FRIDs classes compared to previous research. Additionally, the study explored potential factors associated with the severity of fall injuries. The results showed the severity of fall-related injuries increased with increasing age, a result consistent with that of prior studies [16,17]. Elderly patients, owing to a higher prevalence of comorbidities, had an increased risk of more severe fall-related injuries compared to younger patients.

Notably, our results did not reveal any sex differences in fall-related injuries, which is not consistent with earlier research [18]. Ghosh et al. [18] reported that females were 15.1% more likely to experience falls at a higher severity level. In addition, no significant association was found between FRIDs and fall-related injuries in our study. Earlier research [18,19,20] suggested a positive correlation between opioids and hypoglycemic drugs and fall-related injuries, specifically a three-fold increased risk for insulin users. However, it is important to note that disparities existed in patient inclusion criteria across these studies. For example, some studies included patients aged over 60 years [19] or 70 years [18], while our study involved those aged over 20 years. Additionally, variations in the definitions of fall injury classification were observed. Our hospital adheres to the Taiwan Clinical Performance Indicator, while Lyu et al. followed the Prevention of Falls Network Europe [19], and Ghosh et al. followed the Severity Assessment Code (SAC) [17]. For example, in our study, level 2 was classified as no injury, whereas it was classified as an injury in Lyu et al.’s study [19]. These variations in the fall injury severity classification may contribute to discrepancies in the results.

On the other hand, a systematic review and meta-analysis found no change in the rate of fall-related injuries in persons taking FRIDs [3]. Deprescribing FRIDs did not alter the rate of fall-related injuries (relative risk 0.89, 95% confidence interval 0.57 to 1.39) in outpatients aged 65 and older [3]. Another study showed that the number of antihypertensive medication classes being taken was not linked to an increased risk of serious fall injuries in stroke patients aged 65 and older [21]. These findings suggest that while FRIDs may be associated with the occurrence of falls [9,12,22,23], they may not significantly impact the severity of fall-related injuries.

Mobility impairment resulting from unsteady balance, as the need for standby assistance, was found to be significantly associated with the severity of fall-related injuries [17]. Our results showed that the use of mobility aids reduced the severity of fall-related injuries among high-risk patients. Additionally, our results showed that osteoporosis was associated with more severe fall-related injuries and thus emphasizes the need for treatment of osteoporosis, which is consistent with the findings of prior research [24,25,26,27]. Therefore, providing mobility aids and treating osteoporosis can mitigate the impact of falls, and enhance overall safety in healthcare settings.

This study also has several limitations. As a single-center retrospective study, establishing causal relations and generalizing the findings can be challenging. Despite considering numerous potential risk factors in the analysis, certain confounding factors might not have been assessed. Moreover, certain data, like the Barthel index, could be subject to clinical judgment variations among different assessors.

## 5. Conclusions

While our study did not reveal a significant correlation between the severity of fall-related injuries and the use of FRIDs among hospitalized patients, the results did show that older age and osteoporosis were associated with greater severity of fall-related injuries. The use of mobility aids and treatment of osteoporosis can reduce the severity of fall-related injuries, particularly for high-risk patients. By focusing on interventions such as providing mobility aids to high-risk individuals, healthcare providers can contribute to a safer environment, and reduce the likelihood and severity of fall-related injuries. Further research and multi-center studies would be valuable in confirming and expanding on these findings, ultimately guiding the development of effective fall prevention strategies.

## Figures and Tables

**Table 1:** Demographic and clinical data of 1205 patients with no or mild fall injuries and 26 patients with severe fall injuries.

	No Fall Injury (Level < 3)	Fall Injury (Level ≥ 3)	*p*-Value
Number	1205	26	
Age, years	64.7 ± 15.4	71.9 ± 16.8	0.040
Age ≥ 65	635 (52.7)	18 (69.2)	0.095
Male	734 (60.9)	16 (61.5)	0.948
***Education***			
Primary school	602 (50)	15 (57.7)	0.844
Elementary school	204 (16.9)	3 (11.5)	
High school	263 (21.8)	5 (19.2)	
BMI ^1^	23.6 ± 5.2	22.5 ± 4.4	0.212
Bed restraints	25 (2.07)	0 (0)	0.969
***Number of comorbid diseases at admissions***			
None	591 (49)	11 (42.3)	0.496
1			
≥2	614 (51)	15 (57.7)	
***Comorbid disease at admission***			
Hypertension	566 (47)	9 (34.6)	0.212
Diabetes mellitus	399 (33.1)	7 (26.9)	0.507
Coronary artery disease	220 (18.3)	4 (15.4)	0.707
Liver disease	115 (9.5)	2 (7.7)	0.750
Kidney disease	116 (9.6)	3 (11.5)	0.744
Cancer	270 (22.4)	8 (30.8)	0.313
Dementia	31 (2.6)	1 (3.8)	0.686
Cerebrovascular accident	59 (4.9)	1 (3.8)	0.806
Current smoker	235 (19.5)	6 (23.1)	0.649
Alcohol	47 (3.9)	1 (3.8)	0.989
Surgery history	856 (71)	19 (73.1)	0.820
***Fall risk assessment at admission***			
Unstable gait	619 (51.4)	10 (38.5)	0.193
Dizziness	252 (20.9)	6 (23.1)	0.789
Muscle weakness	305 (25.3)	5 (19.2)	0.480
Mobility aid use	945 (78.4)	16 (61.5)	0.040
Fall history past 1 year	305 (25.3)	8 (30.8)	0.527
Dysphagia	135 (11.2)	3 (11.5)	0.957
***Dependence***			
No dependence (Barthel index = 100)	342 (28.4)	9 (34.6)	0.354
Slight dependence (91–99)	29 (2.4)	1 (3.8)	
Moderate dependence (61–90)	364 (30.2)	3 (11.5)	
Severe dependence (21–60)	347 (28.8)	10 (38.5)	
Total dependence (0–20)	123 (10.2)	3 (11.5)	
***Laboratory data at admission***			
Hemoglobin [g/dL]	11.4 ± 2.5	10.5 ± 2.5	0.082
Prothrombin time [sec]	12.7 ± 3.7	12.1 ± 1.8	0.125
Potassium (K) [mEq/L]	3.9 ± 0.6	4.1 ± 0.7	0.146
Sodium (Na) [mEq/L]	134.6 ± 5.3	133.2 ± 5.2	0.187
***Physiological evaluation***			
Visual impairment	308 (25.6)	8 (30.8)	0.547
Hearing impairment	165 (13.7)	7 (26.9)	0.054
***Admission diagnosis***			
Sleep disorder	53 (4.4)	3 (11.5)	0.084
Cancer	433 (35.9)	9 (34.6)	0.890
Depressive	52 (4.3)	0 (0)	0.279
Kidney disease	164 (13.6)	4 (15.4)	0.794
Stroke	83 (6.9)	1 (3.8)	0.543
Cardiovascular disease	125 (10.4)	4 (15.4)	0.409
***Osteoporosis before admission***			
None	1099 (91.2)	20 (76.92)	0.022
Osteoporosis with drug therapy	51 (4.23)	2 (7.69)	
Osteoporosis without drug therapy	55 (4.56)	4 (15.38)	

^1^ BMI, body mass index. Data presented as mean ± standard deviation, or count (percentage).

**Table 2:** The use of fall-risk-increasing drugs (FRIDs) in 1205 patients with none or mild fall injuries and 26 patients with severe fall injuries.

	No Fall Injury (Level < 3)	Fall Injury (Level ≥ 3)	*p*-Value
Number	1205	26	
***CNS-active medications***			
Opioids	439 (36.4)	9 (34.6)	0.849
Antipsychotics	268 (22.2)	5 (19.2)	0.715
Anxiolytics	353 (29.3)	6 (23.1)	0.490
Hypnotics and sedatives	205 (17)	4 (15.4)	0.827
Antidepressants	113 (9.4)	2 (7.7)	0.770
Antiepileptics	235 (19.5)	4 (15.4)	0.599
***Cardiovascular medications***			
Vasodilators	68 (5.6)	1 (3.8)	0.693
Antihypertensives	48 (4)	0 (0)	0.299
Diuretics	276 (22.9)	2 (7.7)	0.066
Beta blocking agents	244 (20.2)	7 (26.9)	0.403
Calcium channel blockers	249 (20.7)	3 (11.5)	0.254
Renin-angiotensin system inhibitors	265 (22)	6 (23.1)	0.895
Alpha-adrenoreceptor antagonists	145 (12)	1 (3.8)	0.201
***Others***			
Anti-Parkinson drugs/anticholinergic agents	31 (2.6)	0 (0)	0.407
Drugs used in diabetes	364 (30.2)	7 (26.9)	0.718
Anti-inflammatory and antirheumatic products, non-steroids (NSAIDs)	119 (9.9)	4 (15.4)	0.354
Contact laxatives	550 (45.6)	10 (38.5)	0.467
Proton pump inhibitors (vonoprazan excluded)	463 (38.4)	8 (30.8)	0.427
**FIRDs use**			
No	114 (9.46)	3 (11.54)	0.731
Yes	1091 (90.54)	23 (88.46)	
Polypharmacy (drug ≥ 5)	264 (21.9)	5 (19.3)	0.744

Data presented as mean ± standard deviation, or count (percentage).

**Table 3:** Risk factors for severe fall injury (level ≥ 3).

	Crude OR (95% CI)	*p*-Value	Adjusted OR (95% CI)	*p*-Value	Adjusted OR (95% CI)	*p*-Value
Age ≥ 65	1.95 (0.86, 4.44)	0.110	1.49 (0.64, 3.46)	0.356	1.45 (0.63, 3.38)	0.383
Mobility aid use	0.43 (0.2, 0.95)	0.037	0.40 (0.19, 0.88)	0.023	0.40 (0.18, 0.87)	0.021
Hemoglobin level	0.87 (0.75, 1.01)	0.061	0.89 (0.76, 1.04)	0.138	0.89 (0.76, 1.03)	0.127
Potassium (K) level	1.56 (0.96, 2.53)	0.075	1.32 (0.8, 2.18)	0.279	1.31 (0.80, 2.16)	0.286
Sodium (Na) level	0.95 (0.89, 1.02)	0.100	0.96 (0.90, 1.03)	0.295	0.96 (0.9, 1.03)	0.283
Hearing impairment	2.42 (1.02, 5.72)	0.044	1.75 (0.72, 4.27)	0.216	1.8 (0.74, 4.37)	0.191
Sleep disorder	3.21 (1.00, 10.28)	0.050	3.15 (0.98, 10.16)	0.055	3.24 (1.01, 10.4)	0.048
Osteoporosis						
No	1		1		--	--
Yes	3.27 (1.32, 8.11)	0.010	3.02 (1.21, 7.53)	0.018	--	--
Osteoporosis and therapy						
No	1		--		1	
Osteoporosis with drug therapy	2.60 (0.67, 10.08)	0.166	--	--	2.25 (0.59, 8.58)	0.234
Osteoporosis without drug therapy	4.35 (1.50, 12.59)	0.007	--	--	4.23 (1.44, 12.43)	0.009

## Data Availability

The datasets employed and examined in this study can be obtained by contacting the corresponding author, through a reasonable request.
